# Effects of whole body vibration with exercise therapy versus exercise therapy alone on flexibility, vertical jump height, agility and pain in athletes with patellofemoral pain: a randomized clinical trial

**DOI:** 10.1186/s12891-020-03732-1

**Published:** 2020-10-26

**Authors:** Ebrahim Rasti, Zahra Rojhani-Shirazi, Naghmeh Ebrahimi, Mohammad Reza Sobhan

**Affiliations:** 1 Student Research Committee, School of Rehabilitation Sciences, Shiraz University of Medical Sciences, Shiraz, Iran; 2 Department of Physical Therapy, School of Rehabilitation Sciences, Shiraz University of Medical Sciences, Shiraz, Iran; 3 Rehabilitation Sciences Research Center, Shiraz University of Medical Sciences, Shiraz, Iran; 4 Department of Orthopedic Surgery, Shahid Sadoughi University of Medical Sciences, Yazd, Iran

**Keywords:** Patellofemoral pain, Exercise therapy, Whole body vibration, Flexibility, Agility

## Abstract

**Background:**

Patellofemoral pain (PFP) is the most prevalent orthopedic problem in active young adults. Due to its multifactorial etiology, a variety of therapeutic measures have been adopted to treat PFP, including exercise therapy, electrotherapy, and manual therapy. It has also been suggested that whole body vibration (WBV) can improve neuromuscular function in persons with knee problems. The aim of the present study was to evaluate the effects of adding WBV to routine exercise programs on flexibility, vertical jump height, agility and pain in athletes with PFP.

**Methods:**

Twenty-four male athletes with PFP were randomized into two groups of WBV + exercise (*n* = 12) or exercise only (*n* = 12). Participants received their interventions during 4 consecutive weeks (12 sessions). Pain intensity, flexibility and agility were assessed respectively as score on a numerical rating scale, the sit-and-reach test, and a modified T-test, and vertical jump height was measured to the nearest centimeter. The tests were done before and after the interventions, and the results were compared between the two groups. Independent t-tests and paired t-tests were used for between- and within-group comparisons, respectively.

**Results:**

After the interventions, all variables for vertical jump height, flexibility, agility and pain intensity improved significantly in both groups (*p* < 0.05). The flexibility test showed significantly greater improvement in the WBV + exercise group (*p*<0.001), whereas for vertical jump height, agility and pain intensity, there were no statistically significant differences between groups (*p*>0.05).

**Conclusions:**

The present findings showed that exercise therapy with and without WBV can significantly decrease pain and increase agility, vertical jump height and flexibility in athletes with PFP. Adding WBV to routine exercise therapy, however, can augment the effects of the latter on flexibility.

**Trial registration:**

IRCT, IRCT20090831002391N39. Registered 7 February 2018, https://en.irct.ir/search/result?query=IRCT20090831002391N39.

## Background

The term patellofemoral pain (PFP) refers to diffuse retropatellar or peripatellar pain which is exacerbated by physical activities including squatting, prolonged sitting, and ascending and descending stairs [1, 2]. This musculoskeletal condition is known to be the most prevalent orthopedic problem in active young adults, afflicting about 26% of young athletes [2]. It was reported that about 30% of referrals to sports medicine clinics are due to PFP [3]. The prevalence of PFP in Iranian female athletes has been reported as 16.74% [4]. In addition to pain, PFP may entail other symptoms such as crepitus, impaired knee proprioception and functional deficits, which in turn can decrease athletes’ dexterity and agility, and prevent them from achieving optimal performance during sports activities [5].

Numerous studies have reported reduced flexibility in the quadriceps, gastrocnemius, soleus and hamstring in athletes with PFP [6–8]. Moreover, PFP can lead to muscle weakness in the knee extensors, hip abductors and external rotators, as well as in the hamstring [6, 9, 10]. Although the etiology of PFP is not yet completely known, it can be attributed to a variety of neuromusculoskeletal factors including joint laxity, leg length discrepancy, angular or rotational deformities in the lower extremities, patellar dyskinesia, flat foot, increased Q-angle, quadriceps weakness, reduced lower limb flexibility, overuse injuries, and muscle imbalance in the quadriceps, hamstrings, tensor fascia lata and gluteus medius [11, 12].

Flexibility is an important factor in injury prevention and sports performance in athletes [13–16]. Decreased hamstring flexibility was found to be associated with a reduction in vertical jump height in athletes and with the occurrence of PFP [8, 17, 18]. Earlier studies reported several significant differences in flexibility measures between people with and without PFP. Flexibility may be decreased in lower limb muscles (hamstring, quadriceps, gastrocnemius) among people with PFP [6–8, 18, 19]. Poor hamstring flexibility may cause more need of force production of quadriceps or a slight knee flexion during physical activities that these two phenomenons lead to more joint reaction force in patellofemoral joint [8]. In addition vertical jump was reportedly impaired by PFP [17, 20].

A combination of agility, fitness, aerobic and anaerobic capacity, and muscular capabilities is needed for professional sport activity [21]. Agility, balance and coordination are three key factors for precise, rapid movements, and these three factors are affected by proprioception [22], which was found to be impaired in PFP [23]. Training to improve proprioception can increase agility, quickness and acceleration in the performance of sports activities [24, 25]. On the other hand, balance impairment has been found in persons with PFP, and given the obvious relationship between balance and agility, improvements in balance can contribute to improvements in agility [26–28]. Because of strong direct relationships among agility, proprioception and balance, agility may be impaired in PFP. In light of these previous findings, we focused on agility, flexibility and vertical jump height, as well as pain, as the outcomes of interest in the present study.

According to earlier research, several rehabilitation therapies have been suggested to treat PFP including exercise therapy, neuromuscular training and manual therapy. In relation to the factors that contribute to PFP, a combination of stretching and strengthening exercises (especially combinations of exercise therapy with lower-limb stretching) was found to effectively improve symptoms in patients with PFP [19, 29–31].

A technique that has recently gained popularity for managing knee problems is transmitting mechanical oscillations to the body by means of vibrating platforms [32]. It has been suggested that body vibration can induce a range of physiological changes at different levels, and improve neuromuscular function through postural control strategies, tonic vibration reflexes, and muscle tuning mechanisms [32]. Whole body vibration (WBV) has been reported to improve pain and proprioception in patients with knee osteoarthritis [33]. Furthermore, there is evidence that WBV is effective in enhancing the physical performance of healthy athletes, in terms of muscle force, agility, flexibility, vertical jump height and sprinting [34–36]. To the best of our knowledge, however, only one study to date has tested WBV as a management option for PFP [37]. The dearth of trials focusing on this option led us to evaluate the effects of adding WBV to routine exercise programs on pain and physical performance in athletes with PFP. We hypothesized that WBV would augment the therapeutic benefits of exercise therapy. Thus the aim of the present study was to compare the effects of exercise training with and without WBV on flexibility, vertical jump height, agility, and pain in athletes with PFP.

## Methods

### Study design

This was a double-blind parallel randomized clinical trial in which the outcome assessor was unaware of the group allocation of the participants. Patients were aware of the existence of two different groups, but did not know whether they had been assigned to the treatment or control group.

### Participants

Twenty-four male athletes with a diagnosis of PFP as confirmed by an orthopedist were recruited from patients who were referred to the physiotherapy clinic of Yazd Sports Medicine Board, Iran. PFP was diagnosed on the basis of clinical examination. The sample size was calculated on the basis of agility measurements reported in a previous related study (α = 0.05, power = 80, 95% CI) [38].

Patients were included if they had unilateral patellofemoral pain which was aggravated during at least two of these activities: running, hopping, kneeling, squatting, prolonged sitting, and ascending and descending stairs [39]. An additional criterion was a positive result in at least one of the following: patellar apprehension test, vastus medialis coordination test, or eccentric step-down test [40]. Performing sports which require jumping and leaping, for at least three 2-h sessions per week, and an increase in Kujala score from 50 to 80 were other inclusion criteria for this trial [41].

The exclusion criteria were contraindications for WBV (kidney stone disease, diabetes, cardiopulmonary disease, recent fractures, acute edema, acute disk herniation, using a heart pacemaker, epilepsy), as well as any history of patellar dislocation or subluxation, previous hip, knee or ankle surgery, and any other conditions that may cause anterior knee pain, such as tibiofemoral pathologies [30, 42, 43]..

An ethical approval code was provided by the local medical ethics committee (IR.SUMS.REC.1396.138) and this research was registered in the Iranian Registry of Clinical Trials (IRCT20090831002391N39). Written informed consent was obtained from all participants before they received the interventions. Then an online randomization application (www.randomization.com) was used to randomly assign the patients to receive WBV plus exercise training (*n* = 12) or exercise training only (*n* = 12) by permuted block randomization with a block size of four. For group concealment we used a sealed envelope. The allocation ratio was 1:1 (intervention group: control group). An orthopedist diagnosed PFP and enrolled participants in the study, then an expert physiotherapist assigned participants to the intervention groups and trained them. Patients were recruited between February 16 and May 4, 2018. This study was designed and carried out in compliance with CONSORT guidelines.

### Interventions

#### Whole body vibration

The participants stood on the WBV platform in a squatting position with 30 degrees of knee flexion. Patients in the experimental group received 12 sessions of WBV during four consecutive weeks (3 sessions/week), and each session included 2 sets of 60-s training with a 30-s interval between sets. The frequency and amplitude of WBV (FitVibe Excel pro, GymnaUnighy NV, Bilzen, Belgium) were set at 50 Hz and 4 mm, respectively. Whole body vibration was applied during the same sessions as exercise therapy, with a 15-min rest between the two treatments [44, 45].

#### Exercise therapy

Patients of both groups received 4 weeks of exercise therapy (3 sessions of 45 to 60 min/week) in two phases, as follows:

Phase 1 (1st and 2nd weeks):

- 3 min warm-up on a stationary bike
- Quadriceps setting (2 sets, 10 repetitions, 10 s hold), supine straight-leg raises (SLR) (3 sets, 10 repetitions, 10 s hold), side-lying SLR (3 sets, 10 repetitions, 10 s hold), single-leg stance (3 sets, 30 s hold)
- Self-stretches of the Hamstring (30 s hold, 3 repetitions), quadriceps (30 s hold, 3 repetitions), and calf muscles (30 s hold, 3 repetitions)

Phase 2 (3rd and 4th weeks):

- 3 min warm-up on a stationary bike
- Self-stretches of the iliotibial band (30 s hold, 5 repetitions), hamstring (30 s hold, 5 repetitions), quadriceps (30 s hold, 5 repetitions), and calf muscles (30 s hold, 5 repetitions)
- Quadriceps setting (2 sets, 15 repetitions, 10 s hold)
- Dynamic exercises: single-leg cable machine exercises in flexion (3 sets, 10 repetitions), extension and abduction directions (3 sets, 10 repetitions), bilateral mini-squat (3 sets, 10 repetitions), and prone-plank exercise (3 sets, 10 repetitions) [46, 47].

During dynamic exercises, the trainer gave verbal feedback to the participants to correct mistake and increase motivation. Exercises were stopped if participants reported any pain in the knee.

### Outcome measures

We assessed flexibility, agility, vertical jump height and pain as outcomes before and after 4 weeks of treatment.

#### Flexibility

Flexibility was assessed with the sit-and-reach test. To perform the test, patients were asked to sit on the floor with their legs stretched out straight ahead, and their bare feet were placed against a box. Then the patients were instructed to lean forward as far as possible and hold the position for 2 s. The examiner pressed the patients’ knees to the floor and measured the distance between the fingertips and toes with a ruler scaled from − 25 (for fingertips before the toes) to + 25 (for fingertips beyond the toes). The test was repeated after a 30-s interval and the highest score was recorded. This test has good reliability, with an intraclass correlation coefficient (ICC) of 0.92 to 0.96) [48, 49].

#### Agility

**Fig. 1:**
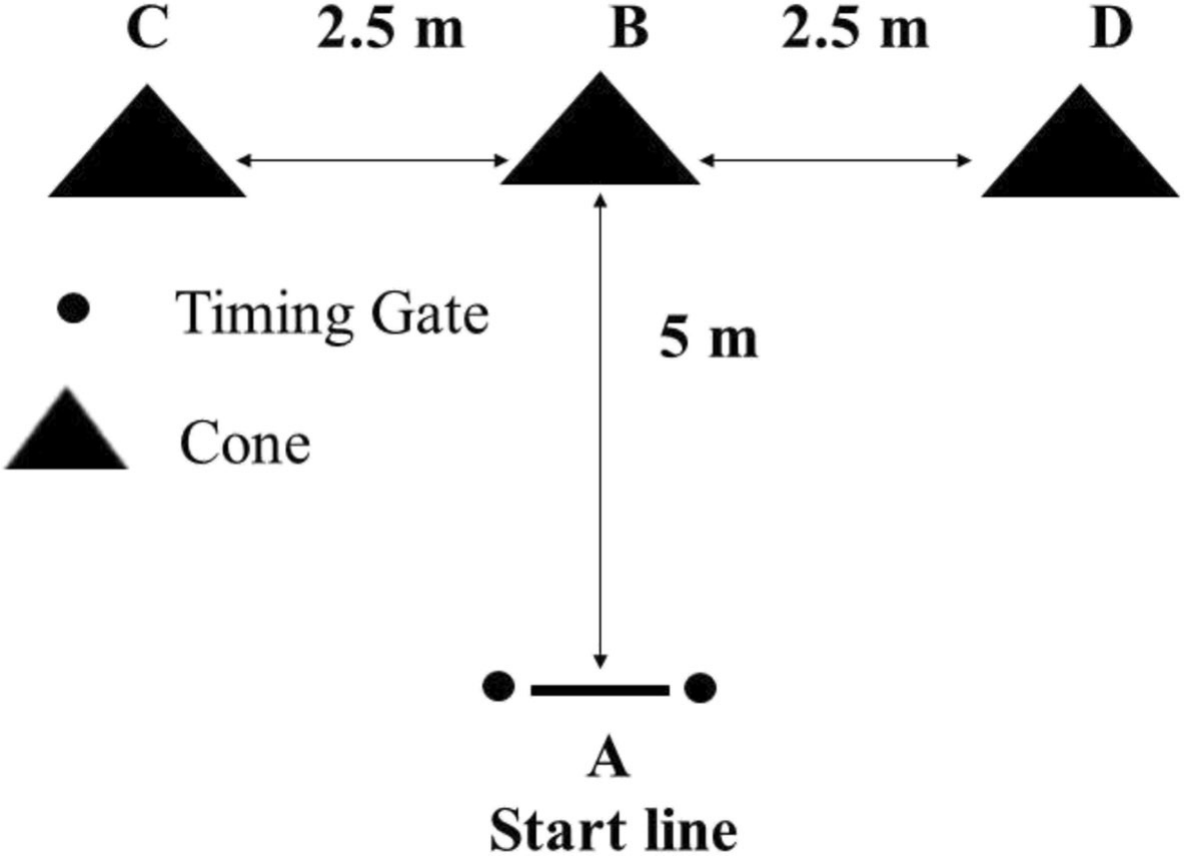
Modified T-test to record agility

Agility was evaluated with a modified T-test, in which the patients negotiated a course between four cones positioned in a T shape, as illustrated in Fig. 1. Distances B-C and B-D were 2.5 m, and distance A-B was 5 m long. The patients started at cone A, sprinted forward to cone B, side-shuffled to each side and then shuffled back to cone B, and returned to cone A as fast as possible, in the following sequence: A, B, C, B, D, B, A. The time needed to complete the test was recorded with a stopwatch. The test was repeated after 3 min in the opposite direction of side-shuffling (A, B, D, C, B, A), and the better score of the two trials was recorded. The reliability of this test has been shown in athletes (ICC: 0.92 to 0.95) [50]. The minimal detectable change for T-test was reported as 1.10 s [51].

#### Vertical jump height

For the vertical jump test, the patients stood next to a wall and extended their arm upward as high as possible. The examiner marked the point of patients’ fingertips on the wall. Then patients were asked to jump vertically as high as possible, and the examiner marked the highest point touched on the wall. The distance between the two marked points was measured in centimeters. This trial was repeated three times and the highest score was recorded. Patients were allowed to countermove their arms before jumping [52]. According to previous studies, a 2–6 cm increase in jump height may be cause a significant change in the jump test [53]. This test has shown good reliability (ICC: 0.93 to 0.97) [50].

#### Pain

Pain intensity was evaluated after the patients ascended and descended a 25-cm step. Pain was measured with a 0-to-10 linear numerical rating scale (NRS), in which 0 equaled “no pain” and 10 indicated “the worst imaginable pain”. This scale was shown to be reliable, valid and sensitive (ICC: 0.67 to 0.82) [54–56]. The minimally clinically important difference for NRS was reported as 1.7 points [57].

### Statistical analyses

The data were analyzed with the Statistical Package for Social Sciences (SPSS), version 20 (IBM Inc., Chicago, IL, USA). The one-sample Kolmogorov–Smirnov test was used to verify normality of the distribution of variables. The values for all variables showed a normal distribution, so parametric tests, i.e. the independent t-test and paired t-test, were used for between- and within-group comparisons, respectively. A *p* value of < 0.05 was considered the significance level for all statistical comparisons. The effect size for comparisons of the results between the two groups was calculated with Cohen’s d: $\frac{M_1-{M}_2}{SD_{pooled}}$ . Effect sizes of 0.2 were considered small, 0.5 medium, 0.8 large, and 1.3 very large [58, 59].

## Results

**Fig. 2:**
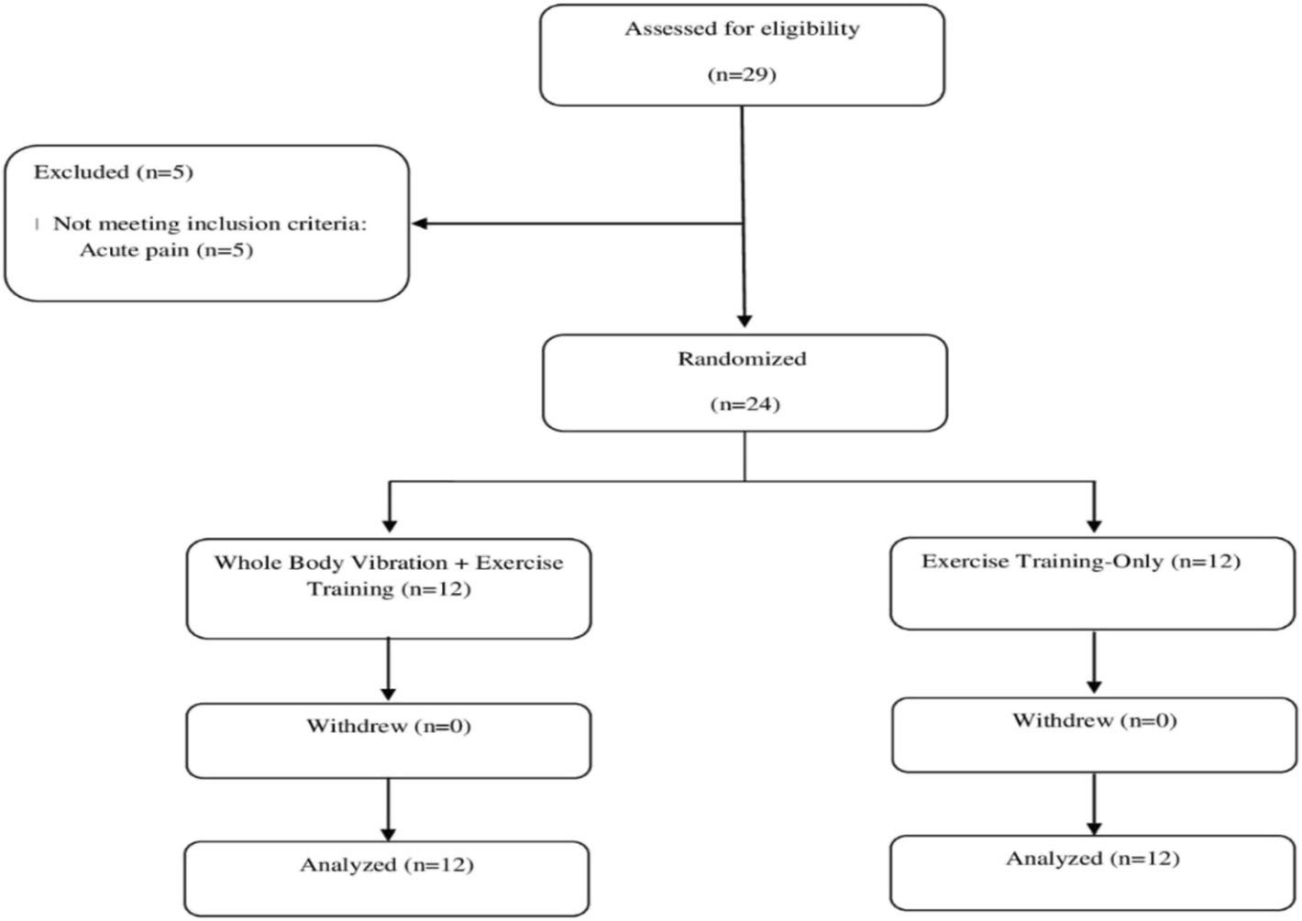
Flow chart of the study

Figure 2 shows the flow chart for this study.

According to Table 1, the two groups were well-matched at baseline regarding their demographic characteristics and baseline values of the outcome measures.

**Table 1:** Demographic data of participants and baseline values for vertical jump, flexibility, agility, and pain intensity

Variable	WBV ^a^ + Exercise group (*n* = 12) (mean ± SD ^b^)	Exercise-only group (*n* = 12) (mean ± SD ^b^)	*p-value*
Age (years)	25.91 ± 5.16	24.16 ± 5.21	0.418
Height (cm)	175.91 ± 6.00	177.50 ± 6.17	0.531
Weight (kg)	74.41 ± 5.85	76.66 ± 5.72	0.352
BMI ^c^ (kg/m^2^)	24.01 ± 0.78	24.31 ± 0.	0.981
Kujala score (0–100)	66.16 ± 5.84	64.91 ± 5.96	0.609
Vertical jump (cm)	46.66 ± 3.52	47.83 ± 3.85	0.447
Flexibility (cm)	19.16 ± 2.51	20.08 ± 2.27	0.359
Agility (s)	7.18 ± 0.41	7.19 ± 0.24	0.948
Pain (0–10)	5.83 ± 0.83	6.08 ± 0.99	0.512

^a^ Whole body vibration, ^b^ Standard deviation, ^c^ Body mass index

As Table 2 shows, all variables for vertical jump height, flexibility, agility and pain intensity improved significantly in both groups (*p* < 0.05).

**Table 2:** Within-group comparisons of vertical jump, flexibility, agility and pain intensity before and after the interventions

Variable		WBV ^a^ + Exercise group (*n* = 12)				Exercise-only group (*n* = 12)			
		Pre	Post	*p*-value		Pre	Post	*p*-value	
Vertical jump (cm)	(mean ± SD ^b^)	46.66 ± 3.52	48.16 ± 3.88	<0.001*		47.83 ± 3.85	49.00 ± 4.17	<0.001*	
	95% CI (Upper bound- Lower bound	48.90–44.42	50.63–45.70			50.28–45.38	51.65–46.34		
Flexibility (cm)	(mean ± SD ^b^)	19.16 ± 2.51	23.91 ± 2.77	<0.001*		20.08 ± 2.27	21.66 ± 2.05	0.001*	
	95% CI (Upper bound- Lower bound)	20.76–17.56	25.68–22.15			21.52–18.63	22.97–20.35		
Agility (second)	(mean ± SD ^b^)	7.18 ± 0.41	6.65 ± 0.41	<0.001*	0.542	7.19 ± 0.24	6.77 ± 0.32	<0.001*	0.596
	95% CI (Upper bound- Lower bound	7.44–6.92	6.91–6.39			7.35–7.03	6.98–6.56		
Pain (0–10)	(mean ± SD ^b^)	5.83 ± 0.83	3.16 ± 1.19	<0.001*	0.655	6.08 ± 0.99	3.83 ± 1.40	<0.001*	0.680
	95% CI (Upper bound- Lower bound	6.36–5.30	3.92–2.40			6.71–5.45	4.72–2.94		

^a^ Whole body vibration, ^b^ Standard Deviation, * Statistically significant change (*p*<0.05)

Based on the between-group comparisons summarized in Table 3, there was a statistically significant difference between the two groups in the results of the flexibility test [mean difference (95% CI): 3.17 (2.07 to 4.27), effect size (95% CI): 2.45 (1.32 to 3.41)]. For vertical jump height [mean difference (95% CI): 0.34 (− 0.48 to 1.16), effect size (95% CI): 0.35 (− 0.47 to 1.15)], agility [mean difference (95% CI): − 0.11 (− 0.36 to 0.14), effect size (95% CI): − 0.3 (− 1.17 to 0.45)], and pain intensity [mean difference (95% CI): − 0.41 (− 1.37 to 0.55), effect size (95% CI): − 0.36 (− 1.15 to 0.46)], however, there were no statistically significant differences between groups (p>0.05).

**Table 3:** Between-group comparisons of pre-post changes in vertical jump, flexibility, agility and pain intensity

Variable	WBV ^a^ + Exercise group (*n* = 12)		Exercise-only group(*n* = 12)		*p*-value	Effect size
	(mean ± SD ^b^)	95% CI (Upper bound- Lower bound	(mean ± SD ^b^)	95% CI (Upper bound- Lower bound		
Vertical jump (cm)	1.50 ± 1.00	2.13–0.86	1.16 ± 0.93	1.76–0.57	0.409	0.35
Flexibility (cm)	4.75 ± 1.48	5.69–3.80	1.58 ± 1.08	2.27–0.89	<0.001*	2.45
Agility (s)	−0.52 ± 0.35	−0.30 - -0.75	−0.41 ± 0.23	−0.26 - −0.56	0.388	−0.37
Pain (0–10)	−2.66 ± 0.88	−2.10 - -3.23	−2.25 ± 1.35	−1.38 - -3.11	0.385	−0.36

^a^ Whole body vibration, ^b^ Standard deviation, * Statistically significant change (*p*<0.05)

## Discussion

The findings of the present study showed that 4 weeks of exercise therapy can improve functional performance in athletes with PFP, and adding WBV can augment the effect of exercise training on flexibility. This far, some studies have investigated the efficacy of WBV on knee performance in older adults [60, 61], patients with knee osteoarthritis [62–64], and patients who have undergone anterior cruciate ligament reconstruction [32, 65]. In patients with PFP, however, only one study has been published, to our knowledge. Corum et al. examined the effects of adding WBV training to home exercises in treating females with PFP, and reported improvements in the total work of knee extensors and less knee pain after WBV training. However, these authors reported no significant difference between groups at 6-month follow-up. In contrast to Corum and colleagues, who used home-based exercises, our patients completed an exercise program under the direct supervision of a well-trained physical therapist. Moreover, Corum et al. tested 24 sessions of WBV with 20–30 min of vibration in each session, whereas our patients received 12 sessions of 2-min WBV [37].

In general, the evidence for the efficacy of adding WBV to conventional exercise training is controversial. Some studies found that WBV added to exercise therapy considerably increased muscle strength and power [66], flexibility [48], muscle cross-section [67], and bone mineral density [68], and also decreased abdominal fat [69]. Delecluse et al. showed that 12 weeks of WBV and resistance training, including static and dynamic knee extensor exercises, significantly increased isometric and dynamic knee extensor strength and counter-movement jump height in untrained females. They attributed the induced strength in knee extensors to the reflexive muscle contraction induced by WBV. However, the study by Delecluse and colleagues included only healthy women, and therefore the effects of WBV on pain and disability were not assessed [66].

A meta-analysis showed that adding WBV to routine exercises led to significantly greater improvements in knee extensor strength and counter-movement jump height compared to the same exercises without WBV, in both younger and older adults [70]. On the other hand, Cochrane et al. showed that 9 sessions of WBV training (5 sets of 1 min per session) did not enhance counter-movement jump height, squat jump height, sprint speed, or agility performance in non-elite athletes [71].

In a meta-analysis published in 2015, Rogan et al. concluded that the addition of WBV to exercise therapy did not induce significantly greater improvements in muscle strength in healthy older adults [72]. This finding was highlighted in another systematic review and meta-analysis in 2016, in which Anwer et al. found no additional effect of WBV on quadriceps muscle strength in patients with knee osteoarthritis [64].

Within-group comparisons in our participants showed reduced pain in both groups, and this reduction can be considered statistically and clinically significant bcause pain decreased more than 1.7 point in both groups. In contrast, the improvements in pain and vertical jump height did not differ significantly between groups, and the effect sizes were small to medium. The jump height changes in our study were less than 2 cm. These improvement may nonetheless be of practical significance.

Norms of sit and reach test for males (aged 20–29) is reported > 30 cm [73] while in our study, the average of this test among the participants was less than 30 cm, which indicates poor flexibility in these patients. In the present study, WBV significantly increased the effect of routine exercise on flexibility in athletes with PFP. The effect size of this improvement was large, and can be considered clinically meaningful and significant in practical terms, thus making it is a noteworthy findings for clinicians. The gains in flexibility after WBV can be attributed to increased blood flow in the muscles, which can raise the temperature of muscle fibers and lead to better muscle flexibility [74]. Moreover, WBV can induce an inhibitory effect in muscles by stimulating Golgi tendon organs and affecting Ia inhibitory interneurons in antagonist muscles, which in turn releases the muscle and improves its flexibility [75, 76]. Vibration also can improve flexibility by affecting the stretch reflex loop. These possible mechanisms are in line with previous findings of improved flexibility after WBV. Jacobs et al. found significant improvements (4.7 cm) in flexibility in healthy adults after the use of WBV in comparison to leg cycling ergometry [77], and Karantrantou et al. concluded that WBV can considerably improve flexibility (3.3 cm improvement) in moderately active females [78]. Fagnani et al. reported 3 cm improvement of flexibility after use of WBV and it was statistically significant [48]. The improvement in flexibility in our study was 4.75 cm. Based on previous studies, abnormal flexibility is related to 2.5 times increase the risk of injury [16]. Poor hamstring flexibility can cause injury in the lower limb, impaired sport performance [17] and in may also cause more need of force production of quadriceps or a slight knee flexion during physical activities that these two phenomenons lead to more joint reaction force in patellofemoral joint [8]. On the other hand, after resistance training, flexibility training is the most important program for injury prevention among athletes [14]. Stretching program for acquisition flexibility is needed in fitness program because normal muscle length can potentially prevent musclutendinous injury during exercise and physical activities [16]. Based on the available evidence WBV by increasing flexibility of hamstring may help athletes with PFP in preventing lower limb injury. It seems that increasing hamstring flexibility and returning it to its normal length should be considered in the treatment of PFP, so that decreased muscle flexibility does not cause injury or decreased function in these patients. Future researches are needed for investigation the other effects of WBV on treatment of individuals with PFP and assessing clinical benefits of this device.

### Study limitations

One of the limitations of the present study was the lack of long-term follow-up to assess the maintenance of the therapeutic effects of WBV. Furthermore, only male athletes were included in this trial; therefore the results may not be generalizable to female patients. An additional factor that should be taken into account is that in the intervention group we added 2 min WBV to exercise therapy, and this may have caused a difference between groups in the volume of training time.

## Conclusion

The findings of the present study show that in athletes with PFP, adding WBV to exercise therapy can lead to a greater improvement in flexibility than exercise therapy alone. This improvement is statistically and clinically significant. But adding WBV to exercise therapy compared to exercise therapy alone, cannot make a significant difference in other functional performance (pain, agility and vertical jump) in patients with PFP. In conclusion, WBV may be implemented in rehabilitation programs for athletes with PFP in order to augment the efficacy of conventional exercise therapy, particularly to improve flexibility as an important factor in athletic performance. Of course, it seems that future studies need to examine the effect of WBV on the treatment of PFP.

## Data Availability

The data file of this study are available from the corresponding author and can be made available to anyone upon reasonable request.

## Abbreviations

PFP: Patellofemoral pain
WBV: Whole body vibration
SLR: Straight-leg raise
NRS: Numerical rating scale
ICC: Intraclass correlation coefficient
